# Vitamin D Status and Depressive Symptoms in Patients Undergoing Hemodialysis: A Cross-Sectional Study

**DOI:** 10.3390/nu18193141

**Published:** 2026-09-24

**Authors:** Nathalia Passos Moura, Djiovana Zanini, Larissa David Soares, Myrella Rosales Teixeira, Caroline Helen Santos Gonçalves Mazala, Eduarda Neutzling Drawanz, Thiago Falson Santana, Isabela Thurow Lemes, André Quincozes-Santos, Carlos Alberto Gonçalves, Mariana Bonati de Matos, Aline Longoni, Diogo Onofre Souza, Adriano Martimbianco de Assis

**Affiliations:** 1 Graduate Program in Health and Behavior, Clinical Neurosciences Laboratory, Catholic University of Pelotas (UCPel), Gonçalves Chaves Street, 373, Room 324C, Pelotas 96015-560, RS, Brazilmariana.matos@ucpel.edu.br (M.B.d.M.);; 2 Graduate Program in Biological Sciences, Biochemistry, Institute of Basic Health Sciences, Federal University of Rio Grande do Sul (UFRGS), Porto Alegre 90035-003, RS, Brazil

**Keywords:** chronic kidney disease, hemodialysis, vitamin D deficiency, 25-hydroxyvitamin D, depressive symptoms, Beck Depression Inventory-II

## Abstract

Background/Objectives: Depressive symptoms are highly prevalent among patients undergoing maintenance hemodialysis. This study investigated the association between vitamin D status and depressive symptom burden in adults undergoing maintenance hemodialysis. Methods: This cross-sectional study included 91 patients treated at a university hospital in southern Brazil. Depressive symptoms were assessed using the Beck Depression Inventory-II (BDI-II), analyzed as a continuous outcome. Serum 25-hydroxyvitamin D [25(OH)D] was measured by chemiluminescence, and deficiency was defined as <20 ng/mL. Linear regression with HC3 robust standard errors was used, adjusting for age, sex, socioeconomic status, antidepressant or anxiolytic use, and vitamin D supplementation. Results: Serum 25(OH)D was available for 90 participants, of whom 19 (21.1%) were vitamin D deficient. Mean BDI-II scores were higher in deficient than non-deficient participants (18.4 ± 12.0 vs. 12.4 ± 9.0). Vitamin D deficiency was associated with a 6.00-point higher BDI-II score in the crude model (95% CI 0.07–11.94; *p* = 0.047) and a 5.05-point higher score after adjustment (95% CI 0.02–10.07; *p* = 0.049). In an extended model including sun exposure, hemodialysis duration, and social support, the estimated difference remained similar (β = 4.69; 95% CI −0.16–9.55; *p* = 0.058), although precision remained limited across the sensitivity analyses. The estimate was attenuated after adjustment for sleep quality, and no linear association was observed when 25(OH)D was modeled continuously. Conclusions: Vitamin D deficiency was associated with greater depressive symptom burden. However, the association was imprecise, attenuated after sleep adjustment, and absent when 25(OH)D was modeled continuously; causality cannot be established.

## 1. Introduction

Chronic kidney disease (CKD) represents a major global public health challenge, with increasing prevalence and substantial impacts on morbidity, mortality, and quality of life [1,2]. Among patients with advanced CKD, those undergoing maintenance hemodialysis are particularly vulnerable to a broad range of metabolic, physical, and neuropsychiatric complications [3,4,5,6,7]. Depressive symptoms are especially common in this population and are associated with poorer quality of life, reduced treatment adherence, hospitalization, and increased mortality [5,6,7]. This burden is also evident in Brazil. In a study conducted in Pelotas, southern Brazil, depressive symptoms were identified in 48% of patients undergoing hemodialysis using the Beck Depression Inventory (BDI), whereas another Brazilian study involving 200 hemodialysis patients reported a prevalence of depression of 29%, illustrating the substantial burden of depressive symptoms as well as the variability associated with different populations and assessment strategies [8,9].

Depressive symptoms in patients with CKD are multifactorial and reflect a complex interaction between biological, psychological, behavioral, and social factors [5,6,7]. Chronic inflammation, accumulation of uremic toxins, metabolic disturbances, high comorbidity burden, impaired physical functioning, and the demanding nature of dialysis treatment may all contribute to depressive symptoms [5,6]. Within this context, potentially modifiable biological factors have received increasing attention. Vitamin D status is particularly relevant because vitamin D deficiency is highly prevalent among patients with advanced CKD and those receiving hemodialysis [10]. Reduced sunlight exposure, dietary restrictions, impaired cutaneous synthesis, and disturbances in vitamin D metabolism associated with advanced kidney disease may contribute to reduced circulating concentrations of 25-hydroxyvitamin D [25(OH)D] [10].

Beyond its established role in mineral and bone metabolism, vitamin D has been increasingly investigated for its potential involvement in brain function and mood regulation [11,12,13,14]. Vitamin D receptors and vitamin D-related metabolic pathways are present within the central nervous system, and experimental evidence suggests that vitamin D may influence neuroinflammatory signaling, neurotrophic processes, neurotransmitter synthesis, calcium-dependent signaling, and neuronal resilience [12,13,14]. These mechanisms provide biological plausibility for an association between impaired vitamin D status and depressive symptoms. Nevertheless, biological plausibility alone does not establish a causal relationship, particularly because vitamin D status may also reflect behavioral and clinical characteristics closely related to depression, including reduced outdoor activity, limited sunlight exposure, nutritional status, chronic disease severity, and treatment-related factors.

Clinical evidence regarding the relationship between vitamin D status and depressive symptoms in kidney disease remains heterogeneous. Previous observational studies have reported associations between vitamin D deficiency and depressive symptoms in patients with CKD [11]. More recently, a large propensity score-matched cohort study involving patients with CKD found that vitamin D deficiency was associated with a higher subsequent risk of major depression, with the association persisting during follow-up [15]. In patients undergoing hemodialysis specifically, Yavuz et al. reported lower 25(OH)D concentrations among patients with depressive symptoms and an association between vitamin D deficiency and depressive symptoms after multivariable adjustment [16]. However, evidence from intervention studies is less consistent. A randomized double-blind trial involving 746 dialysis patients with vitamin D insufficiency and depressive symptoms found that high-dose vitamin D_3_ supplementation did not significantly reduce depressive symptom severity in the overall study population [17]. Taken together, these findings suggest an association between vitamin D status and depressive symptoms but do not establish that vitamin D deficiency directly causes depressive symptoms or that supplementation necessarily improves them.

Several methodological factors may contribute to the discrepant findings across studies, including differences in population characteristics, definitions of vitamin D deficiency, assessment of depressive symptoms, vitamin D supplementation, psychotropic medication use, sunlight exposure, socioeconomic conditions, dialysis-related characteristics, and other clinical or psychosocial factors. Furthermore, much of the available evidence has relied on categorical definitions of depressive status rather than the full range of symptom burden. Evaluating depressive symptoms across their full range may provide complementary information while avoiding information loss from dichotomization.

Therefore, the present study aimed to investigate the association between vitamin D status and depressive symptom burden, assessed using the Beck Depression Inventory-II (BDI-II), in patients undergoing maintenance hemodialysis in southern Brazil. We additionally evaluated whether the association was maintained after accounting for relevant sociodemographic, clinical, treatment-related, behavioral, and psychosocial factors.

## 2. Methods

### 2.1. Study Design and Setting

This cross-sectional study was conducted in the hemodialysis unit of São Francisco de Paula University Hospital, Catholic University of Pelotas (UCPel), Pelotas, Rio Grande do Sul, southern Brazil. Data collection was performed in February 2025. The study was approved by the Research Ethics Committee of the Catholic University of Pelotas under protocol number 7.297.849. The reporting of this study followed the Strengthening the Reporting of Observational Studies in Epidemiology (STROBE) recommendations for cross-sectional studies [18].

### 2.2. Participants

Patients receiving maintenance hemodialysis at the study center were recruited by convenience sampling during the data collection period. Participants were eligible if they were aged 18 years or older, were receiving hemodialysis for CKD, and received treatment through the Brazilian Unified Health System (Sistema Único de Saúde; SUS). Pregnant patients and patients undergoing peritoneal dialysis were excluded.

A total of 91 participants were enrolled. Complete BDI-II data were available for all participants. Serum 25-hydroxyvitamin D [25(OH)D] measurements were available for 90 participants; one participant had missing laboratory data. The number of observations included in each analysis was reported.

### 2.3. Sociodemographic and Clinical Characteristics

Sociodemographic and clinical information was obtained using a structured questionnaire administered during the study assessment. Variables included age, sex, self-reported skin color, educational attainment, socioeconomic status, current antidepressant or anxiolytic use, previous diagnosis of depression, vitamin D supplementation, daily sun exposure, and duration of hemodialysis treatment. Educational attainment was categorized as less than complete high school and complete high school or higher, using completion of Brazilian Ensino Médio (upper secondary education, equivalent to high school) as the cutoff. One participant who did not provide educational information was treated as having missing data. Current antidepressant or anxiolytic use, vitamin D supplementation, and daily sun exposure were recorded as binary self-reported variables (yes/no). For sun exposure, the questionnaire did not capture duration, time of day, body area exposed, or sunscreen use. Detailed information on the formulation, dose, and duration of vitamin D therapy, use of active vitamin D analogues, specific psychotropic agents, medication doses, or treatment duration was not systematically available.

Socioeconomic status was assessed using the Brazilian Economic Classification Criterion (Critério de Classificação Econômica Brasil) developed by the Brazilian Association of Research Companies (ABEP) [19]. For the multivariable analyses, the ABEP score was analyzed as a continuous variable to preserve the information contained in the original score and avoid unnecessary categorization.

### 2.4. Assessment of Depressive Symptoms

Depressive symptoms were assessed using the Beck Depression Inventory-II (BDI-II), a 21-item self-report questionnaire assessing depressive symptomatology over the preceding two weeks. Each item is scored from 0 to 3, yielding a total score ranging from 0 to 63, with higher scores indicating greater depressive symptom burden.

The Brazilian Portuguese version of the BDI-II has demonstrated satisfactory reliability and validity for the assessment of depressive symptoms [20,21].

The BDI-II total score was defined as the primary outcome in the present analysis and was treated as a continuous variable to retain the full information provided by the instrument and avoid the loss of statistical power associated with dichotomization.

For descriptive and secondary analyses, participants were also classified according to conventional BDI-II symptom-severity categories: minimal (0–13), mild (14–19), moderate (20–28), and severe (29–63). Analyses based on severe depressive symptoms (BDI-II ≥ 29) were considered exploratory.

### 2.5. Assessment of Vitamin D Status

Blood samples were obtained by the hemodialysis nursing team during routine monthly blood collection using the participants’ vascular access. Samples were processed and transported to the supporting clinical laboratory according to routine procedures.

Serum 25(OH)D concentrations were measured by chemiluminescence and expressed in ng/mL. Of the 91 participants, 90 had valid serum 25(OH)D measurements.

Vitamin D was evaluated using two complementary approaches. For the primary exposure, participants were classified as vitamin D deficient when serum 25(OH)D concentrations were <20 ng/mL, whereas concentrations ≥20 ng/mL were classified as non-deficient. This threshold is consistent with commonly used clinical definitions of vitamin D deficiency and with Brazilian reference recommendations [22].

The <20 ng/mL cutoff was selected as a clinically interpretable definition of vitamin D deficiency rather than as a dialysis-specific biological boundary. Because published CKD and hemodialysis studies have used heterogeneous 25(OH)D thresholds, the categorical analysis was complemented by a continuous 25(OH)D model so that interpretation would not rely exclusively on a single cutoff.

In a sensitivity analysis, 25(OH)D concentration was also modeled as a continuous variable to evaluate whether depressive symptom burden showed evidence of a linear association with vitamin D concentration.

All valid serum 25(OH)D measurements were included in the analyses.

### 2.6. Additional Covariates

Potential covariates were defined according to their clinical and conceptual relevance to the relationship between vitamin D status and depressive symptoms rather than according to statistical significance in univariable analyses.

The primary adjustment set included age, sex, socioeconomic status (continuous ABEP score), current antidepressant or anxiolytic use, and vitamin D supplementation.

Additional clinical, behavioral, and psychosocial variables evaluated in extended or sensitivity analyses included daily sun exposure, duration of hemodialysis treatment, previous diagnosis of depression, social support, and sleep quality.

Social support and sleep quality were assessed using the corresponding domains of the Kidney Disease Quality of Life Short Form (KDQOL-SF), whose Brazilian Portuguese version has been culturally adapted and validated for patients with end-stage kidney disease [23].

Previous diagnosis of depression was considered in sensitivity analyses rather than simultaneously incorporated into the primary adjustment set because of its close conceptual relationship with current psychotropic treatment and depressive symptom burden.

Sleep quality was examined only in sensitivity analyses because sleep disturbance may be related to vitamin D status but also overlaps conceptually with the depressive phenotype captured by the BDI-II. Consequently, adjustment for sleep could introduce construct overlap and potential overadjustment.

### 2.7. Statistical Analysis

Statistical analyses were performed using IBM SPSS Statistics for Windows, version 27.0 (IBM Corp., Armonk, NY, USA). Continuous variables were summarized as mean and standard deviation or median and interquartile range, according to their distributions, whereas categorical variables were described using absolute and relative frequencies. Distributional characteristics were assessed by graphical inspection and descriptive measures.

The primary outcome was the total Beck Depression Inventory-II (BDI-II) score, analyzed as a continuous variable. The primary exposure was vitamin D deficiency, defined as serum 25-hydroxyvitamin D [25(OH)D] concentrations <20 ng/mL. BDI-II scores were initially summarized according to vitamin D deficiency status using descriptive statistics.

The association between vitamin D deficiency and depressive symptom burden was subsequently evaluated using linear regression. Regression coefficients (β) represent the estimated mean difference in BDI-II score between vitamin D-deficient and non-deficient participants. The adjusted model included age, sex, socioeconomic status assessed using the continuous ABEP score, current antidepressant or anxiolytic use, and vitamin D supplementation. Covariates were selected based on clinical and conceptual relevance rather than on statistical significance in univariable analyses.

To account for heteroscedasticity, HC3 heteroscedasticity-consistent standard errors were used to obtain 95% confidence intervals and *p*-values. Multicollinearity was assessed using variance inflation factors.

As sensitivity analyses, serum 25(OH)D was modeled as a continuous variable, and additional models examined the influence of sun exposure, duration of hemodialysis, previous diagnosis of depression, social support, and sleep quality. These analyses were considered exploratory.

For secondary descriptive analyses, participants were classified according to conventional BDI-II severity categories (minimal, mild, moderate, and severe). The proportion of participants with severe depressive symptoms (BDI-II ≥ 29) was compared according to vitamin D deficiency status using Fisher’s exact test. No multivariable logistic regression was performed for the severe-symptom analysis.

Missing data were handled using complete-case analysis for the variables included in each analysis. Effect estimates are presented with 95% confidence intervals and two-sided *p*-values. Statistical significance was conventionally defined as *p* < 0.05; however, interpretation emphasized effect magnitude and precision rather than statistical significance alone.

## 3. Results

### 3.1. Participant Characteristics

A total of 91 patients undergoing maintenance hemodialysis were enrolled in the study. Serum 25-hydroxyvitamin D [25(OH)D] measurements were available for 90 participants, who constituted the primary analytical sample. The mean age was 54.8 ± 15.2 years, 45 participants (50.0%) were women, and 57 (63.3%) self-identified as White (Table 1).

Educational attainment was available for 89 participants; 57 (64.0%) had less than complete high school and 32 (36.0%) had completed high school or higher. The median duration of hemodialysis was 32 months [IQR 12–60] (Table 1).

Current antidepressant or anxiolytic use was reported by 28 participants (31.1%), 18 (20.0%) reported a previous diagnosis of depression, 19 (21.1%) reported vitamin D supplementation, and 57 (63.3%) reported daily sun exposure (Table 1).

The mean serum 25(OH)D concentration was 31.1 ± 13.4 ng/mL. Nineteen participants (21.1%) were classified as vitamin D deficient (<20 ng/mL), whereas 71 (78.9%) were non-deficient (Table 1). The mean BDI-II score in the analytical sample was 13.6 ± 10.0, with a median of 11.0 [IQR 7.0–20.5] (Table 1).

Participants with vitamin D deficiency had higher depressive symptom scores than non-deficient participants. The mean BDI-II score was 18.4 ± 12.0 among vitamin D-deficient participants and 12.4 ± 9.0 among non-deficient participants. Corresponding median scores were 14.0 [IQR 10.0–24.5] and 11.0 [IQR 7.0–17.0], respectively (Table 1).

### 3.2. Association Between Vitamin D Deficiency and Depressive Symptom Burden

In the crude linear regression model, vitamin D deficiency was associated with an estimated 6.00-point higher BDI-II score compared with non-deficiency (β = 6.00; 95% CI 0.07 to 11.94; *p* = 0.047) (Table 2).

After adjustment for age, sex, socioeconomic status assessed using the continuous ABEP score, current antidepressant or anxiolytic use, and vitamin D supplementation, the magnitude of the association was slightly attenuated. Vitamin D-deficient participants had an estimated 5.05-point higher BDI-II score than non-deficient participants (β = 5.05; 95% CI 0.02 to 10.07; *p* = 0.049) (Table 2).

Within the adjusted model, increasing age was associated with lower BDI-II scores (β = −0.15 per year; 95% CI −0.24 to −0.05; *p* = 0.003), as was a higher ABEP socioeconomic score (β = −0.34 per point; 95% CI −0.60 to −0.08; *p* = 0.011). Current antidepressant or anxiolytic use was associated with higher BDI-II scores (β = 5.20; 95% CI 0.10 to 10.29; *p* = 0.046). No clear associations were observed for sex (β = 0.86; 95% CI −3.55 to 5.27; *p* = 0.703) or vitamin D supplementation (β = −2.44; 95% CI −6.77 to 1.90; *p* = 0.271) (Table 2).

The crude model explained 6.1% of the variability in BDI-II scores (R^2^ = 0.061), whereas the adjusted model explained 26.6% (R^2^ = 0.266; adjusted R^2^ = 0.213) (Table 3).

### 3.3. Sensitivity Analyses

The magnitude of the association between vitamin D deficiency and depressive symptom burden remained relatively consistent when additional clinical, behavioral, and psychosocial variables were considered, although the precision of the estimates varied (Table 4).

In the extended model additionally including daily sun exposure, duration of hemodialysis, and social support, vitamin D deficiency was associated with an estimated 4.69-point higher BDI-II score (95% CI −0.16 to 9.55; *p* = 0.058; *n* = 87) (Table 4).

When a previous diagnosis of depression was added to the primary adjusted model, vitamin D deficiency was associated with an estimated 4.71-point higher BDI-II score (95% CI 0.20 to 9.22; *p* = 0.041) (Table 4).

The association was attenuated after additional adjustment for sleep quality, with an estimated difference of 2.39 BDI-II points between vitamin D-deficient and non-deficient participants (95% CI −2.45 to 7.22; *p* = 0.333) (Table 4).

When serum 25(OH)D was modeled as a continuous variable, no evidence of a linear association with depressive symptom burden was observed. Each 10-ng/mL increase in serum 25(OH)D was associated with an estimated 0.40-point difference in BDI-II score (95% CI −1.00 to 1.80; *p* = 0.578) (Table 4).

### 3.4. Secondary Analyses According to BDI-II Severity

Among the 90 participants with available 25(OH)D measurements, 56 (62.2%) had minimal depressive symptoms, 11 (12.2%) had mild symptoms, 15 (16.7%) had moderate symptoms, and 8 (8.9%) had severe symptoms (Supplementary Table S1).

Severe depressive symptoms were observed in 4 of 19 vitamin D-deficient participants (21.1%) compared with 4 of 71 non-deficient participants (5.6%; Fisher’s exact *p* = 0.058) (Supplementary Table S1).

## 4. Discussion

In this cross-sectional study of patients undergoing maintenance hemodialysis, vitamin D deficiency was associated with a greater depressive symptom burden. Participants with serum 25(OH)D concentrations below 20 ng/mL had BDI-II scores approximately six points higher than non-deficient participants in the crude analysis and approximately five points higher after adjustment for age, sex, socioeconomic status, current antidepressant or anxiolytic use, and vitamin D supplementation (Table 2). The magnitude of this association remained broadly similar across sensitivity models that additionally considered sun exposure, hemodialysis duration and social support, or previous diagnosis of depression, although confidence intervals indicated limited precision (Table 4). Importantly, the finding depended on how vitamin D status was represented: no evidence of a linear association was observed when serum 25(OH)D was analyzed continuously, and adjustment for sleep quality substantially attenuated the categorical association (Table 4). Taken together, these findings support only a cautious interpretation and do not demonstrate a general dose-response or causal relationship.

Our findings are broadly consistent with previous observational studies linking vitamin D deficiency to depressive symptoms in populations with kidney disease. Jhee et al. reported a higher prevalence of depression among vitamin D-deficient patients with CKD and an association that persisted after multivariable adjustment [11], although that study used a substantially lower threshold for vitamin D deficiency (≤10 ng/mL) and included patients across CKD stages rather than specifically focusing on maintenance hemodialysis. More directly comparable to our population, Yavuz et al. evaluated 140 patients undergoing hemodialysis and found lower 25(OH)D concentrations among participants with depressive symptoms, with vitamin D deficiency associated with depression after adjustment [16]. More recently, Chen et al. reported an association between vitamin D deficiency and subsequent major depression in a large propensity score-matched CKD cohort [15]. Although these studies differ substantially in design, population, vitamin D thresholds, and depression assessment, they support further investigation of vitamin D status as a correlate of mental health in CKD.

An important feature of the present study is that the observed association depended on how vitamin D status was represented. Although participants classified as vitamin D deficient had greater depressive symptom burden, serum 25(OH)D modeled continuously was not associated with BDI-II score (Table 4). Thus, the categorical and continuous models are compatible with a possible nonlinear or threshold-related pattern rather than a linear dose-response relationship. However, given the relatively small sample and only 19 vitamin D-deficient participants, these data do not establish a biological threshold at 20 ng/mL. The apparent contrast may also reflect sample variability or the consequences of categorization. Larger studies using flexible nonlinear models are needed before a threshold effect can be inferred.

Biological plausibility for a relationship between vitamin D status and depressive symptoms includes potential effects on neurotrophic signaling, neurotransmission, and neuroinflammatory and oxidative pathways [24,25,26,27,28,29]. However, these mechanisms derive largely from experimental and non-dialysis evidence and should not be interpreted as demonstrating causality in the present population. Depressive symptoms in advanced CKD are multifactorial, with inflammation, oxidative stress, uremic toxicity, comorbidity burden, and treatment-related stress contributing to mental health outcomes [30,31,32,33,34].

Reverse causation represents an equally plausible explanation for at least part of the observed association. Individuals with greater depressive symptom burden may spend less time outdoors, engage in less physical activity, experience changes in diet or appetite, and consequently have lower vitamin D status. In the present study, an extended model additionally including daily sun exposure, hemodialysis duration, and social support yielded an estimate of similar magnitude (Table 4). However, daily sun exposure was available only as a binary self-reported measure and did not capture exposure duration, time of day, body surface area, sunscreen use, ultraviolet intensity, or outdoor physical activity. Accordingly, this variable provides only limited control for behavioral determinants of vitamin D status. The cross-sectional design does not allow determination of whether vitamin D deficiency preceded depressive symptoms or resulted partly from behavioral changes associated with them.

The distinction between association and causation is also supported by intervention studies. In a randomized, double-blind trial involving 746 dialysis patients with vitamin D insufficiency and depression, high-dose vitamin D_3_ supplementation did not produce an overall significant reduction in depressive symptom severity, despite effectively addressing vitamin D status [17]. Evidence from randomized trials outside dialysis populations has likewise been heterogeneous, and systematic reviews have not established a consistent antidepressant effect of vitamin D supplementation [35]. Thus, our findings should not be interpreted as evidence that vitamin D supplementation would improve depressive symptoms in hemodialysis patients. Rather, they identify vitamin D deficiency as a clinical characteristic associated with greater symptom burden that warrants further longitudinal investigation.

Sleep appears to be particularly relevant to the interpretation of our findings. Participants with vitamin D deficiency had poorer sleep scores (Table 1), and additional adjustment for the KDQOL sleep domain reduced the estimated difference in BDI-II scores from approximately five points to 2.39 points, with a confidence interval that included the null (Table 4). Previous research in hemodialysis patients has demonstrated relationships among 25(OH)D status, depressive symptoms, and sleep disturbance; in a study of 121 patients, lower 25(OH)D and higher depressive symptom scores were associated with poorer sleep quality [36]. Poor sleep is itself highly prevalent in the hemodialysis population; a recent meta-analysis of 69 studies and 14,998 patients estimated a pooled prevalence of approximately 64% [37]. Importantly, the BDI-II total score includes a sleep-related item (Item 16). Adding an external sleep scale therefore introduces partial construct overlap between the outcome and covariate and raises the possibility of circularity and statistical overadjustment. This overlap may have contributed to the attenuation of the vitamin D coefficient. The sleep-adjusted model should consequently be viewed as a sensitivity analysis rather than as a preferred causal model or as evidence that sleep simply confounds the association.

Other findings reinforce the multifactorial nature of depressive symptoms in hemodialysis. Higher socioeconomic status, reflected by the continuous ABEP score, was associated with lower BDI-II scores, whereas current antidepressant or anxiolytic use was associated with higher scores (Table 2). The latter should not be interpreted as an adverse effect of psychotropic treatment; patients with more persistent or clinically recognized depressive and anxiety symptoms are inherently more likely to receive such medications, making confounding by indication a likely explanation. Social support also warrants consideration in this population. Recent evidence from patients receiving hemodialysis has shown that greater perceived social support is associated with lower depressive and anxiety symptoms, less fatigue and pain, better sleep, and better mental health-related quality of life [38]. These observations support considering psychological, socioeconomic, behavioral, and treatment-related factors alongside biological biomarkers when investigating depressive symptoms in CKD.

Age was also inversely associated with BDI-II score in the adjusted model (Table 2). Although this secondary finding should be interpreted cautiously, it is consistent with age-related differences reported in dialysis populations. In a recent multicenter study of 783 hemodialysis outpatients, depressive symptoms were less common among patients aged 65 years or older than among younger patients (45.5% vs. 59.7%) [39]. Such patterns may reflect differences in coping, expectations, and psychological adaptation to chronic treatment, although cohort, survival, and clinical selection effects are alternative explanations. Longitudinal studies are needed to clarify whether increasing age itself is associated with improved psychological adaptation during maintenance hemodialysis.

The present study has several strengths. Depressive symptoms were analyzed primarily using the full BDI-II score, thereby preserving information across the entire symptom range rather than relying solely on a dichotomized severe-symptom outcome. Vitamin D status was examined both according to a clinically established deficiency threshold and as a continuous exposure. The analyses also considered several factors particularly relevant to the vitamin D–depressive symptom relationship in hemodialysis, including vitamin D supplementation, psychotropic medication use, sun exposure, socioeconomic status, hemodialysis duration, previous diagnosis of depression, social support, and sleep quality. In addition, all valid serum 25(OH)D measurements were retained, and adjusted models were estimated using heteroscedasticity-consistent standard errors.

Several limitations should nevertheless be considered. First, the cross-sectional design precludes assessment of temporality and causal inference, and reverse causation cannot be excluded. Second, participants were recruited by convenience sampling from a single hemodialysis center, limiting generalizability to other dialysis populations. Third, the sample was relatively small and only 19 participants met the definition of vitamin D deficiency, resulting in limited precision for some estimates and restricting the stability of sensitivity and categorical analyses. Fourth, although the revised models incorporated several relevant covariates, residual confounding remains possible because information on dialysis adequacy (Kt/V), anemia and iron status, serum albumin, systemic inflammatory markers, detailed nutritional status, and other comorbidities was not available for the present analysis. Fifth, serum 25(OH)D was measured at a single time point and may not reflect long-term vitamin D status. Data were collected in February, during summer in southern Brazil, which may also limit generalization across seasons. Sixth, the BDI-II measures depressive symptom burden and should not be interpreted as establishing a clinical diagnosis of major depressive disorder. Finally, sun exposure, vitamin D supplementation, antidepressant or anxiolytic use, previous diagnosis of depression, and other clinical characteristics were based on available clinical or self-reported information. Sun exposure was recorded only as a binary variable, and reliable information on vitamin D formulation, dose, duration, active vitamin D analogues, specific psychotropic agents, medication doses, and treatment duration was not available; these limitations may have introduced exposure or treatment misclassification.

## 5. Conclusions

In conclusion, vitamin D deficiency (<20 ng/mL) was associated with a greater depressive symptom burden among patients undergoing maintenance hemodialysis, with an estimated difference of approximately five BDI-II points after adjustment for relevant sociodemographic and treatment-related factors (Table 2). However, the association was imprecisely estimated, was attenuated after accounting for sleep quality, and was not observed when serum 25(OH)D was modeled continuously (Table 4). These findings may be compatible with a nonlinear pattern concentrated at deficient concentrations, but the present sample is insufficient to establish a threshold effect, causality, or therapeutic benefit. Larger longitudinal studies incorporating repeated vitamin D measurements and more comprehensive assessment of dialysis-related, nutritional, inflammatory, behavioral, and mental health factors are needed to clarify the nature of this relationship.

## Figures and Tables

**Table 1:** Characteristics of the analytical sample according to vitamin D status.

Characteristic	Total (*N* = 90)	Non-Deficient ≥ 20 ng/mL (*n* = 71)	Deficient < 20 ng/mL (*n* = 19)
Age, years	54.8 ± 15.2	54.5 ± 14.6	56.1 ± 17.6
Female sex, *n* (%)	45 (50.0)	32 (45.1)	13 (68.4)
White skin color, *n* (%)	57 (63.3)	45 (63.4)	12 (63.2)
Educational attainment, *n* (%)			
Less than complete high school	57 (64.0)	44 (62.9)	13 (68.4)
Complete high school or higher	32 (36.0)	26 (37.1)	6 (31.6)
ABEP socioeconomic score	23.3 ± 7.5	23.6 ± 7.8	21.8 ± 6.4
Hemodialysis duration, months	32 [12–60]	32 [14–60]	30 [8–78]
Current antidepressant/anxiolytic use, *n* (%)	28 (31.1)	22 (31.0)	6 (31.6)
Previous diagnosis of depression, *n* (%)	18 (20.0)	13 (18.3)	5 (26.3)
Vitamin D supplementation, *n* (%)	19 (21.1)	17 (23.9)	2 (10.5)
Daily sun exposure, *n* (%)	57 (63.3)	47 (66.2)	10 (52.6)
KDQOL social support score	100.0 [66.7–100.0]	100.0 [66.7–100.0]	66.7 [66.7–100.0]
KDQOL sleep score	70.6 ± 24.5	74.5 ± 22.3	55.8 ± 27.1
Serum 25(OH)D, ng/mL	31.1 ± 13.4	35.3 ± 11.7	15.2 ± 3.9
BDI-II total score	13.6 ± 10.0	12.4 ± 9.0	18.4 ± 12.0
BDI-II, median [IQR]	11.0 [7.0–20.5]	11.0 [7.0–17.0]	14.0 [10.0–24.5]

Data are presented as mean ± standard deviation, median [interquartile range], or *n* (%), as appropriate. Educational attainment was available for 89 participants because one participant did not provide this information (non-deficient group: *n* = 70; deficient group: *n* = 19). Hemodialysis duration was available for 87 participants. ABEP, Brazilian Association of Research Companies; BDI-II, Beck Depression Inventory-II; KDQOL, Kidney Disease Quality of Life; 25(OH)D, 25-hydroxyvitamin D.

**Table 2:** Linear regression models for the association between vitamin D deficiency and BDI-II total score.

Variable	β	95% CI	*p*-Value
**Model 1—Crude model (*N* = 90)**			
Vitamin D deficiency (<20 ng/mL)	6.00	0.07 to 11.94	0.047
**Model 2—Adjusted model (*N* = 90)**			
Vitamin D deficiency (<20 ng/mL)	5.05	0.02 to 10.07	0.049
Age, per year	−0.15	−0.24 to −0.05	0.003
Female sex	0.86	−3.55 to 5.27	0.703
ABEP socioeconomic score, per point	−0.34	−0.60 to −0.08	0.011
Current antidepressant/anxiolytic use	5.20	0.10 to 10.29	0.046
Vitamin D supplementation	−2.44	−6.77 to 1.90	0.271

β coefficients represent the estimated mean difference in BDI-II total score. For vitamin D deficiency, the reference category is serum 25(OH)D ≥ 20 ng/mL; for sex, male; for antidepressant/anxiolytic use and vitamin D supplementation, no use. Models were estimated using ordinary least-squares regression with heteroscedasticity-consistent HC3 standard errors. Covariates were selected on clinical and conceptual grounds.

**Table 3:** Model statistics.

Model	*N*	R^2^	Adjusted R^2^
Model 1	90	0.061	0.050
Model 2	90	0.266	0.213

**Table 4:** Sensitivity analyses of the association between vitamin D status and BDI-II total score.

Analysis	*N*	β for Vitamin D	95% CI	*p*-Value
Primary adjusted model	90	5.05	0.02 to 10.07	0.049
Extended model: + sun exposure, hemodialysis duration and social support	87	4.69	−0.16 to 9.55	0.058
Primary model + previous diagnosis of depression	90	4.71	0.20 to 9.22	0.041
Primary model + KDQOL sleep score	90	2.39	−2.45 to 7.22	0.333
25(OH)D modeled continuously, per 10-ng/mL increase ^1^	90	0.40	−1.00 to 1.80	0.578

^1^ The continuous 25(OH)D model was adjusted for the same covariates included in the primary adjusted model. Sensitivity analyses were considered exploratory.

## Data Availability

The data that support the findings of this study are available from the corresponding author upon reasonable request.

## Supplementary Materials

The following supporting information can be downloaded at: https://www.mdpi.com/article/10.3390/nu18193141/s1. Table S1: Distribution of BDI-II symptom severity categories according to vitamin D status.
