# Supplementary material for: Vitamin D Status and Depressive Symptoms in Patients Undergoing Hemodialysis: A Cross-Sectional Study
Source: Nutrients. 2026 Sep 24;18(19):3141. doi: 10.3390/nu18193141 (PMC13667252; doi:10.3390/nu18193141)
Supplement: Supplementary file 1 [file nutrients-18-03141-s001.zip › nutrients-4554938-supplementary.pdf]

Supplementary Materials

Table S1. Distribution of BDI-II symptom severity categories according to vitamin D status.

BDI-II category	Non-deficient (n=71)	Vitamin D deficient (n=19)
Minimal, 0–13	47 (66.2%)	9 (47.4%)
Mild, 14–19	10 (14.1%)	1 (5.3%)
Moderate, 20–28	10 (14.1%)	5 (26.3%)
Severe, ≥29	4 (5.6%)	4 (21.1%)

Severe depressive symptoms were observed in 4 of 19 vitamin D-deficient participants (21.1%) and 4 of 71 non-deficient participants (5.6%). Fisher's exact test, $p=0.058$. BDI-II, Beck Depression Inventory-II.
